# The Second COVID-19 Wave Is a Tsunami With Aftershocks: Mucormycosis Ire as Seen in a Tertiary Care Hospital in Uttarakhand, India

**DOI:** 10.7759/cureus.47358

**Published:** 2023-10-20

**Authors:** Dimple Raina, Ranjana Rohilla, Shiwang Patwal, Ajay Pandita

**Affiliations:** 1 Microbiology, Shri Guru Ram Rai Institute of Medical and Health Sciences, Dehradun, IND; 2 Community Medicine, Shri Guru Ram Rai Institute of Medical and Health Sciences, Dehradun, IND

**Keywords:** pandemic, covid-19, black fungus, zygomycetes, mucormycosis

## Abstract

Background: During the spread of severe acute respiratory syndrome-coronavirus 2 (SARS-CoV-2) or the coronavirus disease 2019 (COVID-19) pandemic in recent times, an upsurge of invasive fungal infections (IFIs) such as mucormycosis was witnessed by many countries like India. This COVID-19-associated mucormycosis (CAM) has presented as a menace to the already creaking health infrastructure. Clinical manifestations, risk factors, and end clinical outcomes varied for every other region/country. The aim of this study is to delineate and analyze plausible clinical and epidemiological factors and associated predictors of CAM in suspected patients presenting to a tertiary care hospital in Uttarakhand, India, during the second wave of COVID-19 in India.

Material and methods: A total of 200 cases of suspected post‑COVID-19 mucormycosis were enrolled. Data were collected taking into account parameters such as hospitalization and ICU admissions during the episode of COVID-19 infection, steroid/antibiotics/oxygen requirement, and comorbidities such as diabetes mellitus, hypertension, or any chronic illness and outcome.

Results: Participants diagnosed with CAM using KOH examination and fungal culture were analyzed in the study (n=46). The median age of patients included was 48, 73.9% were males, and 26% were females. The major predisposing factor was found to be diabetes mellitus type 2. Our work suggests that the mean duration between COVID-19 episodes and CAM was 11.86 days with a significant statistical association. Oxygen requirement and imprudent use of steroids/antibiotics were also allied with mucormycosis.

Conclusion: The burden of such IFIs is expected to be unveiled in tropical countries during pandemics such as COVID-19, which lead to immunosuppression in masses post-treatment. Comorbidities such as diabetes, chronic kidney disease, and hypertension add to the risk of acquiring other infectious disease. Such times require competent healthcare professionals such as diagnosticians, physicians, and surgeons who are skilled to manage such IFIs timely.

## Introduction

Severe acute respiratory syndrome coronavirus 2 (SARS-CoV-2), the etiological agent for coronavirus disease 2019 (COVID-19), has been incriminated as a trigger for the emergence of an extensive spectrum of opportunistic bacterial and fungal infections [1]. During the second wave in early 2021, COVID-19 created massive havoc across India battering the country’s health infrastructure. Adding woes to the demanding situation was mucormycosis, an invasive fungal infection frequently labeled as ‘black fungus’ [2,3]. The term ‘black fungus’ is an erroneous term that was glorified by media and by various research bodies confounding the agents of dematiaceous fungi with those causing mucormycosis [4,5]. The causative agents of care are the mucormycetes, a group of molds that are angio-invasive, and include genera Rhizopus, Mucor, Rhizomucor, Cunninghamella, and Absidia [1]. The surge of this fungal infection in post-recovery COVID-19 cases was, to such an extent, that mucormycosis was affirmed as an epidemic in several states of India and a potentially notifiable disease [3]. The swift dissemination rate and high morbidity and mortality rates caused by the fungus further led to the deterioration of the already flared-up situation [6,7].

Mucormycosis can be divided into six types based on anatomic location: rhinocerebral, pulmonary, cutaneous, gastrointestinal, disseminated, and uncommon presentations (endocarditis, osteomyelitis, peritonitis, and pyelonephritis). The nasal cavity and paranasal sinuses (clinical presentations resemble those of sinusitis) are involved predominantly, but rapid dissemination to orbital and intracranial sites particularly in immunocompromised hosts could radically worsen the prognostic outcome [6,7]. Black eschar formation often can also be visualized in such patients involving the nasal cavity or hard palate, but cannot be attributed as the sole distinctive criterion [8]. Broad ribbon-like aseptate hyphae (coenocytic mycelia) and zygospores seen in microscopic examination are good elementary predictors for the presence of mucormycetes. Factors primarily deemed responsible and, apparently, that seemed to assist the germination of Mucorales spores in COVID-19-infected people, were glucocorticoids, low oxygen milieu (hypoxia), deterioration in glucose levels (diabetes, hyperglycemia), metabolic acidosis, diabetic ketoacidosis, augmented serum free iron levels (growth and virulence of pathogenic organisms are influenced by increased ferritin levels), immunosuppression-induced reduction in phagocytosis, lymphopenia caused by COVID-19 infection, the association of comorbidities, mechanical ventilation, and protracted hospital stay [1]. Zinc and iron supplements used rampantly for COVID-19 infection were also believed noteworthy in the causal logarithm of mucormycosis.

The aftermath of the second wave of the COVID-19 pandemic saw a drastic rise in COVID-19-associated rhino-orbital-cerebral mucormycosis (CAROCM) and pulmonary mucormycosis cases [2,9,10]. The lack of ICU beds complexed by the constant augmentation of severely sick patients in the hospital, seemingly deplenishing hospital resources almost everywhere in India, healthcare worker fatigue, and the lack of in-time diagnostic services added to the mortality rates of post-COVID-19 mucormycosis [2]. Timely diagnosis plays a vital role in assessing the progress of the disease with prudent microbiological KOH and/or culture, biopsy, surgical debridement, and management by apposite systemic antifungals enhancing the prognosis and survival of the patient [11]. This study was undertaken to determine the demographic profile and associated risk factors, clinical presentations, and the predominant species of order Mucorales that were incriminated in the study population of our hospital setting.

## Materials and methods

A retrospective observational study was conducted in the Microbiology Department of Shri Guru Ram Rai Institute of Medical and Health Sciences tertiary care hospital for a period of 18 months from May 2021 till November 2022. Approval from the Institutional Ethical Committee (IEC) was obtained for this retrospective study. Since it was a retrospective study, it was exonerated for patient's consent and their names, along with the hospital ID. Numbers were not revealed to protect their privacy. A separate study ID was assigned for each case. Analysis of a total of 200 cases of suspected post-COVID-19 mucormycosis was performed during this study. The inclusion criteria included a) all the cases presenting with nasal and orbital cellulitis, involvement of nasal bridge and facial skin with blackening, black eschar on nasal or palatine mucosa, concomitant nasal bleeding, and palatal ulceration; b) patients with ophthalmic presentations such as proptosis followed by ophthalmoplegia and visual loss; and c) severe presentations such as cerebral involvement and vascular compromise suggesting invasive mucormycosis. These cases were diagnosed as coronavirus-positive or had recovered from COVID-19 and were subject to meticulous evaluation by an otolaryngologist, ophthalmologist, neurosurgeon, and maxillofacial surgeon. Exclusion criteria included non‑COVID patients and HIV patients.

A brief history considering parameters such as hospitalization and ICU requirement during COVID-19 infection, steroid use, requirement for higher generation antibiotics, comorbidities such as diabetes mellitus, hypertension, and any immunosuppressive illness or use of immunosuppressive drugs was taken. Definitive mucormycosis was diagnosed in those patients having attributable clinical presentations with concurrent exhibition of fungi in the tissue or body fluids either by direct microscopy (KOH mount) and/or culture. Computerized tomography and magnetic resonance imaging (MRI) scan findings to know the extent of the disease were also taken into consideration during the diagnosis.

## Results

A total of 46 participants’ data diagnosed with mucormycosis were analyzed in the study. The median age of patients included was 48. Of all the patients, 73.9% (34) were males, and 26% (12) were females. A significant proportion of subjects (i.e., 26 had diabetes mellitus (56.52%), and 14 (30.43%) had hypertension and chronic kidney disease) was reported in 11 (23.9%) patients. The majority of patients (i.e., 19, 41%) presented with facial/periorbital/cheek swelling on the face, followed by severe headache in 11 (23.9%), fever in eight (17.39%), nasal discharge in five (10.86%), and throat pain and cough in three (6.52%). Numbness on the face and drooping of eyelids were seen in two (4.34%) patients. The mean duration between COVID-19 episodes and mucormycosis was 11.86 days (Table 1). Outcome analysis shows mortality in 16 (34.78%) cases, while 30 (65.2%) were discharged with advice to follow up.

**Table 1:** Species of mucormycosis agents isolated in culture (total number=26). The data have been represented as numbers (N) and percentages (%).

Particulars	Number	Percentage (%)
Rhizopus	20	76.9
Rhizomucor	3	11.53
Cunninghamella	3	11.53

All were diagnosed and hospitalized with COVID-19 during the second wave of the pandemic. Based on the computerized tomography scan severity score (CTSS), the majority (n=43, 93.47%) had moderate-to-severe COVID-19 pneumonia. Invasive mechanical ventilation was carried out in the majority of subjects (n=29, 63%), and systemic corticosteroids were administered to 40 (i.e., 87% of all patients). About 43% of patients received one of the two antivirals (i.e., fevipiravir or remdesivir). More than three to four subjects (n=37, 78.7%) received at least one anti-viral medication (remdesivir or favipiravir), and 34 (73.91%) patients received antibiotics. Only a minority (i.e., seven (15.2%)) received tocilizumab monoclonal antibody (Table 2).

**Table 2:** Comparison of KOH mount with culture findings (n=46). The data have been represented as numbers (N) and percentages (%).

	KOH and/or culture positive	KOH and culture positive	KOH positive, culture negative	KOH negative, culture positive	KOH negative, culture negative
Number of cases	34	15	19	11	1
Percentage	73.91%	32.60%	41.30%	23.91%	2.17%

**Figure 1:**
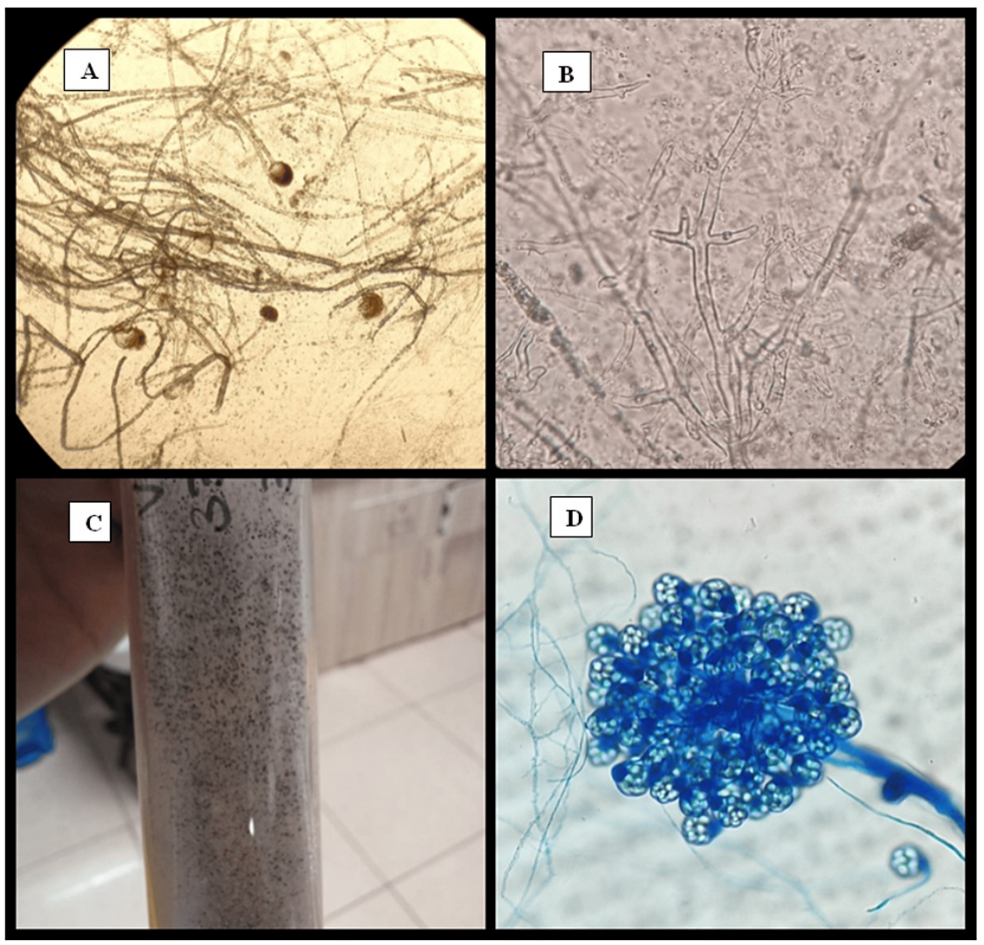
A) and B) Direct tissue KOH microscopy findings showing broad aseptate hyphae and sporangiophores. C) Culture growth of mucormycosis species with salt and pepper appearance. D) Lactophenol cotton blue mount of Cunninghamella species. The image was created by the authors.

Out of all 46 patients, 34 (73.91%) were KOH-positive, and 26 (56.52%) were positive in culture (Tables 3, 4). Figure 1 shows some of the positive findings in KOH examination and culture.

**Table 3:** Comparison of KOH mount with culture findings (n=46). The data have been represented as numbers (N) and percentages (%).

KOH Mount	Culture		Total (%)
	Positive	Negative	
Positive	15	19	34 (73.91%)
Negative	11	1	12 (26.08%)
Total	26 (56.52%)	20 (43.47%)	46

**Table 4:** Severity and management of COVID-19 pneumonia. The data have been represented as numbers (N) and percentages (%). The p value is considered significant at p<0.05. (s)*-significant, (ns)**-non-significant

	Particulars	Number	Percentage (%)	P value
Severity of COVID-19 pneumonia on the basis of the CT severity score	Mild (<7/25)	3	6.52	-
	Moderate (7-18/25)	36	78.2	-
	Severe (>18)	7	15.2	-
Oxygen support	Mechanical ventilation	29	63	P=0.025538 (s)
	Non-invasive respiratory support	14	30.43	
	No oxygen needed	3	6.52	
Steroid use	Use	40	87	P= 0.019036 (s)
	Non -usage	6	13.04	
Antibiotics	Antibiotic use	34	73.91	P= 0.00001 (s)
	Antibiotic non-usage	12	26.08	
Tocilizumab	Use	7	15.2	-
	Non-usage	39	84.7	-
Favipiravir	Use	8	17.39	-
	Non-usage	38	82.6	-
Remdesivir	Use	12	26.08	-
	Non-usage	34	73.09	-
Anticoagulants	Use	24	52.17	-
	Non-usage	22	47.82	-

Speciation of fungi-causing mucormycosis revealed Rhizopus spp. in 76.9% of cases (Table 5).

**Table 5:** Baseline characteristics of patients, predisposing factors, and diagnosis of the patients. The data have been represented as numbers (N) and percentages (%). The p value is considered significant at p<0.05. (s)*-significant, (ns)**-non-significant

Parameter	Particulars	Number (N)	Percentage (%)	P value
Age (in years) n=46	20-30	7	15	-
	31-40	10	21.7	-
	41-50	11	23.9	-
	51-60	10	21.7	-
	61-70	8	17.39	-
Gender n=46	Male	34	73.9	P= 0.3346 (ns)**
	Female	12	26.08	
Predisposing factors	Diabetes mellitus type 2	26	56.52	P= <0.00001 (s)*
	Hypertension	14	30.43	P= 0.286174 (ns)
	Chronic kidney disease	11	23.91	P=1.4152 (ns)
	Hypothyroidism	4	8.69	-
	Seizure disorder	1	2.17	-
	Malignancy	1	2.17	-
Clinical sign and symptoms	Facial/periorbital/cheek swelling on face	19	41	-
	pain/redness in eye/blurring of vision	17	36.9	-
	Dyspnoea	12	26	-
	Severe headache	11	23.9	-
	Fever	8	17.39	-
	Nasal discharge	5	10.86	-
	Throat pain	3	6.52	-
	cough	3	6.52	-
	Numbness on face	2	4.34	-
	Drooping of eyelid	2	4.34	-
	Altered sensorium	2	4.34	-
Duration between COVID and mucormycosis	1-10 days	27	58.69	P=0.000291 (s)
	11-20 days	12	26.06	
	21-30 days	6	37.5	
	>30 days	1	2.17	
Diagnosis of patients	KOH positive	34	73.91	-
	Culture positive	26	56.52	-
Treatment used for post-COVID mucormycosis	Amphoterecin B/posaconazole only	23	50	-
Treatment used for post-COVID mucormycosis	Amphoterecin B/posaconazole+Surgery	23	50	-
Outcome for the patients	Discharge	30	65.2	P=0.000253 (s)

## Discussion

In 2020 during and post the first wave of COVID-19, cases of mucormycosis reported were quite exiguous; however, the second wave in 2021 led to a multifold rise in cases of mucormycosis [12]. The aftermath of the COVID-19 second wave in India inundated acute infections, post-infective pneumonia, and a plethora of opportunistic infections particularly in immunocompromised patients, having comorbidities such as diabetes mellitus type 2, patients having received high-dose (oral) or intravenous steroids, and even those on mechanical ventilation [13].

In the current study, 46 cases were evaluated, and it was observed that 34 (73.9%) males were afflicted, whereas females constituted 12 (26.1%) of the cases showing a male preponderance for the risk of infection. Shaik et al. and Irfan et al. in their studies have also reported a male preponderance of mucormycosis at 80% and 60%, respectively [14,15]. The ratio of male to female was 3:1. The mean age of case subjects was 46.45 ± 13.46 years, with a range spectrum from 24 to 74 years. The data comprise a significant case population from post second wave of the COVID-19 pandemic who had either recovered from the disease or were in the active phase of the disease.

The major predisposing factor was found to be diabetes mellitus type 2 (56.52%), followed by hypertension (30.43%) and chronic kidney disease (23.91%) in our study (P value < 0.00001 for diabetes mellitus: statistical association significant). Concurrent findings have also been reported by Desai et al. (65%) [16] and Singh et al. (80%) [1], wherein diabetes mellitus was acknowledged as the chief comorbidity contributing factor. There is augmented expression of glucose regulatory protein 78 (GRP78) in human endothelial cells, which functions as a receptor for angioinvasion by Mucor spp. glycosylation of transferrin and ferritin caused by amplified blood glucose levels, causing diminished iron-binding capacity and increased free iron levels, a perfect platform for the growth of mucorales [1,17-19].

In the current study, facial, periorbital, and cheek involvement was seen in 41% of cases, followed by the involvement of eyes in 36.9% of the cases. Kumari et al. and Gupta et al. have also reported similar findings; periorbital involvement in 53% and 65%, respectively [6,20]. The mean duration between COVID-19 and the occurrence of mucormycosis was found to be 11.8 days±10.06 days in our research work. In congruence to this, a mean duration of 12 days has also been reported in a study from Pakistan by Irfan et al. and 16 days by Patel et al. [15,21]. However, a longer duration of 20 and 23 days has been reported by Nihara et al. and Ismaiel et al. and Janjua et al., respectively [22-24]. Our work suggests a significant statistical association (p value=0.000291) of the duration between COVID-19 infection and subsequent mucormycosis incidence. The mean interval of time between the diagnosis of COVID-19 and clinical diagnosis of mucormycosis infection holds an imperative level of significance for treating clinicians as cautious watchfulness for the occurrence of mucormycosis, and two to three weeks after abatement of COVID-19 infection is required [25]. During this time, corticosteroid therapy should be rapidly tapered off, and frequent interaction with patients is necessary to keep blood glucose in control. Oxygen support by mechanical ventilation and non-invasive respiratory support was required by 90.43% of the case subjects, which are 63% and 30.43%, respectively. The statistical association between oxygen support and the occurrence of mucormycosis was found to be significant (p value<0.05) in our study. However, the substantiation regarding the role of supplemental oxygen during COVID-19 illness and the ensuing development of COVID-19-associated mucormycosis (CAM) is ambivalent. The present study shows increased oxygen requirement among CAM cases during the COVID-19 pandemic, which has also been reported in studies by Patel et al. and Sen et al. [26,27]. In contrast, such an association has been refuted in studies from Delhi and Pune [28,29]. Unhygienic oxygen delivery practices, increased use of industrial oxygen during the acute wave, usage of contaminated masks, and frequent use of the same mask on different patients for prolonged intervals of time have been attributed as discerning factors for the increment of mucormycosis in COVID-19 patients [30-32].

In this study, 40 (87%) patients diagnosed with mucormycosis were found to have been administered corticosteroids during their COVID-19 infection (P value was found to be statistically significant < 0.05). This is in concurrence with the study conducted by Shaik et al. wherein a similar figure of 87% of patients, who had received steroids during COVID-19, were reported to be suffering from mucormycosis. In yet another study conducted by Kumari et al. [6,14], 80% of patients with mucormycosis were reported to have received steroids for COVID-19 infection. Steroids as an important predisposing factor for CAM have been reported by many other studies from India [1,3,33-37]. The lack of compliance with standard guidelines on steroid usage for COVID-19 has been attributed as one of the important factors for the aggravation of CAM during and following the second wave in India, particularly in prediabetic and diabetic patients where protracted sugar levels in blood resulted in invasive CAM [4,38-41]. Inhibition of phagocytes such as macrophages and neutrophils and a spike in blood sugar levels by corticosteroids seem to be the most conceivable reasons for risk association with CAM.

Usage of antibiotics in 34 (73.91%) cases of CAM with a significant statistical association (p value<0.05) was observed in our research work. Containment of normal bacterial flora by injudicious antibiotic use during COVID-19 management could be one of the contributing factors for fungus establishment. Concurrent with our findings, Langford et al. found that the prevalence of antibiotic use was 74.6% in COVID cases [42]. Calderon-Parra et al. in their study reported antibiotic prescriptions in 78.1% of COVID patients, and 34% of antibiotic prescriptions were found to be inept [43]. Sulis et al. in their study on an interrupted time series analyses of sales volumes of antibiotics in India have estimated that 216.4 million excess doses of antibiotics, and 38.0 million excess doses of azithromycin were prescribed/used until the peak of the first pandemic wave [44].

In this research work, 34 (73.91%) patients were diagnosed as positive by KOH mount wherein broad aseptate/pauciseptate ribbons such as hyphae with random wide-angle branching, sporangia, and rhizoids were seen. Direct microscopy by KOH expedites the early diagnosis of mucormycosis, thereby helping the treating doctors to initiate anti-fungal therapy at the incipient stages of infection [45,46]. Culture positivity was seen in 56.52% of the cases in this study, and lactophenol cotton blue (LPCB) mounts from the colonies revealed Rhizopus spp. as the predominant etiological agent in 76.9% (20) of the cases. Rhizomucor spp. and Cunninghamella spp. each constituted 11.53% (three) of the cases. Hoenigl et al. in their review study from 18 countries on CAM have reported Rhizopus spp. as the most commonly isolated agent from these patients; Rhizopus arrhizus is the most common species in India [47,48]. Similar observations by Rashbi et al. and Muthu et al. corroborate our findings for Rhizopus spp. as the predominant species causing CAM in India [45,49]. Mahalaxmi et al., Rao et al., Sen et al., and Singh et al. have also reported Rhizopus species as the most common, followed by Mucor, Rhizomucor, Lichtheimia, Cunninghamella, and Absidia in their respective studies [27,50-52]. High-temperature conditions can be well-tolerated by members of Mucorales; therefore, hot, dry summers in India with low humidity provide a perfect platform for the growth of these fungi, leading to high prevalence in India. Putting into the picture the perfect concoction of immunosuppression and steroid use during COVID-19, a swaggering increase in mucormycosis cases is no surprise. Improper handling of suspected samples and preceding antifungal drug usage might explain the 41.3% (19) of KOH-positive and culture-negative samples and 2.17% (1) of KOH-negative and culture-negative samples [52-54].

The evidence-based line of treatment is based on a combination of antifungals (liposomal amphotericin B; a dose of 5 mg/kg/day) and aggressive surgical debridement with clear margins if necessary. In the absence of treatment, mortality is high. Regarding the outcome of patients with CAM, the mortality in CAM patients in our study was found to be 34.78% (16), whereas for COVID-19 patients without this fungal infection, the mortality was 11.68%, and the statistical association was found to be significant (p value=0.00025). Watanabe et al. in their study also observed that patients who developed CAM had poorer outcomes than the patients without CAM, though the factors determining a contributory relationship yet remain to be established [3,55].

Finally, there were certain limitations to this study as we did not have a control group, and the strength of the association of many other confounding factors remains to be ascertained. The number of proven mucormycosis cases was limited, and the cases were selected from an assorted population (having a variety of predisposing factors and numerous sites of involvement). Therefore, it was quite intricate to chart out the conclusions from each and every clinical presentation. Additionally, some patients included in our study had received prior COVID-19 treatment before they were admitted to our hospital. Hence, vital information regarding the risk factors could have been omitted, although we tried our best to include those subjects for this study whose comprehensive information was available.

## Conclusions

To summarize, CAM was found to have a strong association with host predisposing factors such as diabetes mellitus, steroid prescription, injudicious antibiotic use, and oxygen support. These risk factors were potentially unveiled as curtain raisers in gaining an insight into the causative cycle of a spike in mucormycosis cases post-second wave, wherein fungi such as Rhizopus and Mucor gained predominance as etiological agents of CAM. Apposite treatment and preventive goals targeting these risk factors evolved to be the prospective intervention areas for averting the ensuing mucormycosis infections post-COVID-19.
